# Tigecycline-induced acute pancreatitis in a renal transplant patient: a case report and literature review

**DOI:** 10.1186/s12879-018-3103-z

**Published:** 2018-05-02

**Authors:** Jinwen Lin, Rending Wang, Jianghua Chen

**Affiliations:** 1 Kidney Disease Center, the First Affiliated Hospital, College of Medicine, Zhejiang University, Qingchun Road, Hangzhou, 310003 China; 2 Key Laboratory of Kidney Disease Prevention and Control Technology, Hangzhou, Zhejiang Province China; 3 The Third Grade Laboratory under the National State, Administration of Traditional Chinese Medicine, Hangzhou, 310003 China

**Keywords:** Tigecycline, Pancreatitis, Adverse events, Kidney transplantation

## Abstract

**Background:**

The purpose of this case report is to increase the awareness of tigecycline-induced pancreatitis specifically in renal transplant patients predisposed to the condition.

**Case presentation:**

A 48-year-old woman developed a donor-derived infection after kidney transplantation, resulting in a ruptured graft renal artery, followed by peritoneal drainage, blood and urine culture infections. Due to multiple drug resistance *Acinetobacter baumannii* cultured from the preservation fluid and blood, she was treated with tigecycline at the 8th post-transplant day combined with other antibiotics. After 15 days of tigecycline treatment, she was observed with recurrent fever and abdominal distension with a rise in pancreatic enzymes. CT scans showed acute pancreatitis with grade D on Balthazar score, no necrosis visible without contrast injection. These facts were sufficient to hint that pancreatitis was slowly becoming prominent. After withdrawal of tigecycline, CT scans showed that exudation around the pancreas were relieved, and blood amylase returned to the normal range in a week.

**Conclusions:**

Clinicians should pay attention to clinical signs and symptoms and the level of serum pancreatic enzymes in order to monitor the development of pancreatitis. If necessary, abdominal CT scans should be performed regularly when given tigecycline.

**Electronic supplementary material:**

The online version of this article (10.1186/s12879-018-3103-z) contains supplementary material, which is available to authorized users.

## Background

Tigecycline was the first member in the glycylcycline class of antibacterial agents to be used clinically. It was approved by the US Food and Drug Administration (FDA) for the treatment of complicated skin and skin-structure infections (cSSSIs). Furthermore, it can be used for the treatment of complicated intra-abdominal infections (CIAI) caused by susceptible Gram-positive, Gram-negative and anaerobic organisms [1]. Tigecycline is a structural derivative of minocycline sharing similar pharmacokinetic properties and adverse effects with tetracyclines. The most common adverse effects associated with tigecycline are nausea, vomiting and diarrhea. Pancreatitis has been found to be associated with tetracycline. However, it was not listed as an adverse drug reaction in the product label when tigecycline was originally approved. A subsequent retrospective cohort analysis and a review of phase 3 and 4 comparative studies of tigecycline have also been performed with mixed conclusions [2, 3]. As tigecycline has a broad spectrum, it has been used as part of the antimicrobial regimen for complicated infections in patients who had received an organ transplant. We report a case of tigecycline-induced acute pancreatitis after kidney transplantation and review the relevant literature.

## Case presentation

**Fig. 1:**
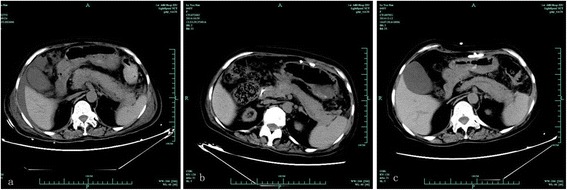
image of CT scan during the the whole course of AP. **a** Twentie-five days post transplantation, the CT scan of abdomen showed pancreatic swelling, peripheral exudate effusion, considered acute pancreatitis (grade D on Balthazar score, no necrosis visible without contrast injection) after tigecycline treatment for 20 days. **b** scan showed a basically normal after tigecycline discontinuation for 14 days. **c** CT reported a normal finding after tigecycline discontinuation for 58 days

A 48-year-old woman with end-stage renal disease (ESRD) due to chronic glomerulonephritis received a kidney transplant from a donor with DCD (donated cardiac death). The kidney was successfully transplanted to the recipient and normal serum creatinine levels were observed after 7 days. On the fourth day after transplantation, the patient was treated with teicoplanin, cefoperazone, sulbactam and etimicin due to the development of multiple drug resistance *Acinetobacter baumannii* in both organ preservation solution and drainage fluid. Tigecycline was administered intravenously at 100 mg for the first dose and was given at 50 mg every 12 h from the eighth day after the operation because of persistent abdominal infection*.* She felt pain at the transplant kidney area and then the blood pressure dropped to 88/61 mmHg on the twentieth day after transplantation. Emergency ultrasound showed two huge hematoma around the graft. Graft pain relieved after emergency treatment including transplanted kidney exploration, renal hematoma removal, renal vascular reconstruction and ureteral re-implantation. The treatment with tigecycline was continued based on the results of the peritoneal drainage, blood and urine culture. The symptoms did not worsen until approximately 15 days after being initially administered. The patient presented with fever, nausea, vomiting and moderate abdominal pain. Physical examination found moderate tenderness in the upper abdomen. Laboratory analyses were remarkable for leukocytosis and the level of lipase raised to 156 U/L. Other results such as serum amylase level was 424 U/L and drainage amylase was 554 U/L, despite the aminotransferase and alkaline phosphatase were within the normal range. CT scans (Fig. 1a) suggested acute pancreatitis (AP) with grade D on Balthazar score, no necrosis visible without contrast injection. There was no sign of dilated biliary ducts according to the abdominal ultrasound examination. Since those findings were considered to be related to drug-induced pancreatitis, it was recommended that tigecycline should be discontinued on the 16th day following exposure. Shortly after tigecycline discontinuation, the patient’s symptoms gradually improved. Blood amylase and lipase returned to baseline levels in a week. CT scans (Fig. 1b) showed a basically normal after tigecycline discontinuation for 14 days. She was discharged from the hospital with a low-fat diet for 3 weeks. One month later, abdominal CT scans on follow-up did not find any abnormalities and showed as normal (Fig. 1c). Throughout the course of treatment, the immunosuppressive regimen was a triple therapy based on recommended doses including tacrolimus, mycophenolate mofetil and prednisone. Serum tacrolimus concentration was maintained at 6–8 ng/ml. The patient received these medications over the next 6 months, with no discomfort and relapse after stopping tigecycline. Timeline of disease was showed in Additional file 1: Figure S1.

## Discussion

Infection from DCD donors is a major challenge in China. A recent study from China showed that 19.4% of donor blood cultures showed blood infection, consistent with the literature that about 5% -11.3% of donors did not find bacteremia on donation. Data from this single-center showed that the incidence of donor-derived bacterial infection was 4.5% [4, 5]. Because this patient’s donor-associated pathogen was multidrug-resistant *Acinetobacter baumannii*, we added tigecycline to the antibiotic regimen based on Chinese expert consensus [6].

Tigecycline is the first member of a new class of antibiotics called glycylcyclines which was licensed by the US FDA in June 2005 for intravenous (IV) use in adults [7]. It inhibits bacterial protein synthesis by binding to the 30S ribosomal subunit with a five-fold higher affinity than tetracycline [8]. Tigecycline is a derivative of minocycline, with a 9-t-butylglycylamido group added to the 1st carbon on the D ring of minocycline and it possesses a broad spectrum range. In 2008, tigecycline received FDA’s approval for the treatment of adult patients with community-acquired bacterial pneumonia [9]. In vitro studies demonstrated that tigecycline exhibits a high level of antimicrobial activity against many common types of respiratory bacteria, including multiple resistant Gram-positive, Gram-negative, anaerobic, as well as multi-drug-resistant (MDR) pathogens, such as methicillin-resistant *Staphylococcus aureus* (MRSA), vancomycin-resistant *Enterococcus* [10], penicillin-resistant *Streptococcus pneumoniae* (PRSP), methicillin-resistant *Staphylococcus epidermidis* (MRSE) and β-lactamase-producing *Haemophilus influenzae* [11].

The most common adverse effects associated with tigecycline are nausea, vomiting and diarrhea. Pancreatitis has been reported that it could be induced by tetracycline, but it was not listed as an adverse drug reaction in the product label when tigecycline was originally approved. Concerns about tigecycline-induced AP have been raised by the clinicians in nearly 10 years [12–14]. Therefore, the former manufacturer of tigecycline, Wyeth, updated the product label including AP as one of the post-marketing adverse events in July 2006 [1]. Interestingly, although tigecycline was registered as a treatment for CIAI, it shoule be used causally when infectious complications were associated with acute pancreatitis because of the possible tigecycline–induced pancreatitis. This exclusion criteria might have emerged due to the similarities between tigecycline and other tetracyclines.

Biliary tract disease (40%) and alcohol exposure (35%) are common causes of AP. Other etiologies include idiopathic pancreatitis, post-endoscopic retrograde cholangiopancreatography, trauma, medications, infection, hypercalcemia, hypertriglyceridemia, tumor and autoimmune diseases. Several drugs are associated with AP. The overall incidence of drug-induced AP is 0.1% ~ 2% [15]. Medications associated with pancreatitis include tetracyclines, isoniazid, macrolides, metronidazole, propofol, angiotensin-converting enzyme inhibitors (ACEI), etc. [16].

The cause of drug-induced pancreatic injury is unknown. By using the classification of Zimmerman originally described for drug hepatotoxicity, drugs associated with tissue-specific injury can be divided into those with intrinsic toxicity in the organ involved and those that cause injury as a result of host idiosyncrasy [17]. As opposed to intrinsic toxicity, idiosyncratic reaction appears to be the mechanism of drug injury in the vast majority of cases. It is known that erythromycin stimulates motilin release which may induce AP by causing spasms of the sphincter of Oddi, leading to an abrupt of hypertension in the pancreatic duct and pancreatitis [18]. Unlike the erythromycin cause the host idiosyncrasy, the mechanism of tetracycline-induced pancreatitis is still unknown. There have been at least three mechanisms hypothesized, including formation of a toxic metabolite, hypertriglyceridemia and a high biliary concentration [12, 19, 20]. Tigecycline is structurally related to minocycline and shares similar pharmacokinetic properties and side effects with tetracyclines [21]. The diagnosis of drug-induced AP is difficult to establish, mainly due to the absence of cause-specific diagnostic tests. Therefore, it is usually based on the following criteria: (1) AP occurs during the administration of a drug. [22] All other common causes are excluded. (3) Symptoms of AP disappear after drug withdrawal. (4) Symptoms recur after a rechallenge of the suspected drug [23]. A total of 10 literatures were collected from the database, which met the above inclusion criteria, involving a total of 12 cases with 13 occasions. [12–14, 16, 21, 24–27]. (Tables 1 and 2) At the same time, the included literature should include the general condition, past history, clinical manifestations of acute pancreatitis, CT scan, daily dose, combination drug therapy, symptom relief time, recovery time, medication time, enzyme recovery time and other information.

**Table 1:** Review of cases report of tigecycline-induced acute pancreatitis --- demographic data and drug characteristics

Author	Country	Number of cases	Year of report	Age of patient	Gender	Indication of tigecycline	Culture of specimens	Duration of tigecycline (days)	Daily dose (mg)	Combination drug	Hitory of liver disease
Glison M	France	1	2008	35	Male	Chronic osteitis complicated by pseudarthritis	*Enterobacter cloacae* with broad-spectrum betalactamase	15	100	Imipenem, amikacin	None
Lipshitz J	Usa	1	2009	64	Female	Prosthetic joint infection	NA	14	100	Levothyroxine,	None
Marshall RS	USA	1	2009	55	Female	Soft Tissue Infection	Enterococcus faecalis, Pseudomonas aeruginosa, and Staphylococcus hominis	14	NA	Pantoprazole, and hydromorphone.	None
Hung WY	USA	1	2009	69	Female	Soft tissue infection/vascular graft infection	Coagulase-negative Staphylococcus, *Staphylococcus epidermidis* and diphtheroids, Clostridium difficile	8	100	Meropenem, vancomycin, clindamycin	None
Prot-Labarthe S	France	1	2010	9	Male	Bacteriemia / arthritis	Enterobacter cloacae producing extended spectrum betalactamase	56	100	Colistin, amikacin and rifampin	None
Otero RS	Mexico	1	2010	27	Female	Acute pneumonia	NA	7	100	Amikacin, oseltamivir	None
Mascarello M	Italy	1	2012	NA	NA	Chronic osteomyelitis	methicillin-resistant *Staphylococcus aureus*, multidrug-resistant Pseudomonas aeruginosa and Acinetobacter baumannii	12	100	Amikacin, propofol	None
Hemphill MT	USA	1st	2015	22	Male	Acute bronchitis	M. chelonae	14	NA	Tobramycin, meropenem, and vancomycin	None
Hemphill MT	USA	2nd	2015	22	Male	Acute bronchitis	M. chelonae	3	NA	Amikacin, clarithromycin	None
Marot JC	Belgium	case 1	2012	64	Male	Soft tissue infection	Staphylococcus aureus	6	100	None	None
Marot JC	Belgium	case 2	2012	58	Male	Soft tissue infection/osteomyelitis	Staphyloccus scleiferi methicillin-resistant and Staphylococcus lugdunensis methicillin-sensitive	8	100	Piperacillin-Tazobactam, Vancomycin	None
Davido B	France	Case 1	2016	70	Male	Pyelonephritis	ESBL *Escherichia coli*	6	100	None	None
Davido B	France	Case 2	2016	50	Female	Femoral osteomyelitis	EBSL *E. coli*,	20	100	Imipenem amikacin.	None

*NA* not available

**Table 2:** Review of cases report of tigecycline-induced acute pancreatitis --- Clinical findings of cases

Author	Onset of symptoms	Clinical manifestation	Amylase/Lipase levels (u/L)	CRP (mg/l)	CT scan	AP severity	Time to symptoms relieved (days)	Time to recovery of enzymes (days)
Glison M	13 days, abdominal pain	Acute abdominal ‘stab-like’ pain	(−)/1000	35	Pancreatic oedema without any necrotic flows (Balthazar Stage 1).	Mild	2	43
Lipshitz J	14 days, epigastric pain	Nausea, vomiting, abdominal pain	806/1406	NA	Mild inflammatory stranding about the duodenum and minimal fluid in the left retroperitoneum.	Mild	3	5
Marshall RS	3 days, uncontrolled emesis	Nausea, vomiting, fever and loss of appetite	180/156	NA	Acute pancreatitis	Mild	2	7
Hung WY	3 days, nausea and vomiting	Persistent and worsening nausea and vomiting, abdominal pain	926/749	NA	NA	NA	3	5
Prot-Labarthe S	14 days, abdominal pain	Abdominal pain, recurrent vomiting	(−)/603	NA	Inflammation involving pancreas and peripancreatic fat without necrosis (Ranson Score 2 and Balthazar stage 2).	Mild	3	5
Otero RS	7 days	Nausea, vomiting, epigastric pain, distention	255/424	NA	Pancreatic enlargement, low density shadow of pancreas tail (grade D on Balthazar score)	Mild	3	12
Mascarello M	12 days	Nausea, vomiting and acute severe upper abdominal pain	312/382	131	Inflammation of the pancreas and peripancreatic fat, necrosis of 40% of the pancreatic gland, peripancreatic stranding, and fluid collection (Balthazar CT severity index 7).	severe	≤10	≤10
Hemphill MT	10 days	Abdominal pain	NA/732	NA	Acute pancreatitis	Mild	6	6
Hemphill MT	3 days	Mild nausea, epigastric tenderness	381/268	NA	Acute pancreatitis	Mild	5	5
Marot JC	6 days, epigastric pain	Nausea, epigastric pain	750/936	NA	An oedematous pancreatitis (grade D on Balthazar score)	Mild	4	18
Marot JC	7 days, abdominal pain on day 8	Nausea, vomiting and loss of appetite	552/1660	NA	Acute pancreatitis, no necrosis visible without contrast injection (grade D on Balthazar score)	Mild	5	4
Davido B	6 days,	Anorexia, vomiting and abdominal discomfort	NA/ 2460	NA	Typical oedematous infiltrate (Balthazar A).	Mild	2	2
Davido B	20 days	Nausea, abdominal discomfort	NA/ 1340	NA	NA	NA	1	1

*CT* Computerized Tomography, *AP* Acute Pancreatitis, *CRP* C-Reactive Protein

Tetracycline-induced AP is closely related to the underlying liver disease in patients [20, 28]. For patients without a history of liver disease, the onset of AP caused by tetracycline for anti-infection therapy ranged from 4 months to 2 years with an average total daily dose of 1083 ~ 1353 mg/d. However, for those patients with severe hepatic dysfunction, the course of tetracycline lasted for 8~ 17 days with an average total daily dose of 1500 ~ 4000 mg/d [29]. Recovery time of amylase and lipase to the normal range was within 13 ~ 21 days. In our review, the administration of tigecycline for the 12 patients was all in accordance with the specification, which indicated that there was no significant correlation between AP and medication. If patients had no history of liver disease, tigecycline-induced acute pancreatitis was more rapid compared to tetracycline. Meanwhile, after withdrawal of tigecycline, the symptoms were relieved more quickly and the time for serum lipase and amylase decline was also shorter.

Twelve patients were given anti-infective drug combination therapy. Moreover, most of the drugs for discontinuation of tigecycline were still used continually. In some cases, propofol increases serum triglyceride levels and may cause AP. However, the patient did not have any hyperlipidemia, and neither of the previous propofol uses caused pancreatitis [30]. There was no evidence that aggravating pancreatitis was associated with propofol combination therapy. Our patient was taking several additional drugs possibly. However, during treatment or ceasing of tigecycline, she has been taking these medications at recommended dose which were associated with pancreatitis and classified as group III (prednisone) and group IV (tacrolimus), respectively (low risk) [18]. Therefore, we do not think that combination therapy in this situation may lead to acute pancreatitis.

The increasing degree of amylase and lipase is not directly related to the severity of AP. Lipshitz J et al. reported the serum lipase and the amylase were as high as 806 UI/L and 1406 UI/L in patients with abdominal pain associated with nausea and vomiting, while CT scans showed duodenal mild inflammation and left posterior abdominal effusion [13]. However, Mascarello M et al. reported that serum lipase and amylase were 382 UI/L and 312 UI/L respectively in patients with similar symptoms [31]. CT scans showed severe pancreatitis inflammation with 40% pancreatic and peripancreatic necrosis and fluid retention, and the Balthazar classification was E level with CTSI score 7 points. It should be noted that the final time to normalize serum lipase and amylase was not directly related to serum lipase and amylase levels.

Finally, tigecycline induced AP is still considered to be a rare phenomenon. However, serum amylase and lipase levels should be closely monitored if any symptomatic abdominal pain suggests AP during treatment. In addition, the mechanism of tigecycline-induced AP and the possibility of cross-reactivity with tetracycline in patients with AP induced by tigecycline should be given more attention.Whether tigecycline increases the risk of pancreatitis in immunosuppressed recipients requires more research data.

## Conclusions

Clinicians should pay attention to clinical signs and symptoms and the level of pancreatic enzymes in the blood in order to monitor the development of pancreatitis. Abdominal CT images should be taken on a regular basis when necessary for the administration of tigecycline.

## Additional file

Additional file 1: **Figure S1.** Timeline. (DOC 70 kb)

## Abbreviations

ACEI: Angiotensin-converting enzyme inhibitors
AP: Acute pancreatitis
CIAI: Complicated intra-abdominal infection
cSSSIs: Complicated skin and skin-structure infections
CT: Computed Tomography
CTSI: CT severity index
DCD: Donation Cardiac Death
ERCP: Endoscopic retrograde cholangiopancreatography
ESRD: End-stage renal disease
FDA: Food and Drug Administration
IV: Intravenous
MDR: Multidrug-resistant
MRSA: Methicillin-resistant *Staphylococcus aureus*
MRSE: Methicillin-resistant *Staphylococcus epidermidis*
PRSP: Penicillin-resistant *Streptococcus pneumoniae*
VRE: Vancomycin-resistant *Enterococcus*
