# Supplementary material for: Tigecycline-induced acute pancreatitis in a renal transplant patient: a case report and literature review
Source: BMC Infect Dis. 2018 May 2;18:201. doi: 10.1186/s12879-018-3103-z (PMC5930510; doi:10.1186/s12879-018-3103-z)
Supplement: Supplementary file 1 — Figure S1. Timeline. (DOC 70 kb) [file 12879_2018_3103_MOESM1_ESM.doc]

**Timeline (Figure S1)**

operation

post-RT

4^th^ day

8^th^ day

21^st^ day

23^rd^ day

24^th^ day

31^st^ day

52^nd^ day

82^nd^ day

She received teicoplanin, cefoperazone and sulbactam and etimicin against the infection.

Tigecycline was added to the antibiotic regimen because of persistent abdominal infection

Discontinuation of Tigecycline.

The symptoms gradually improved.

Moderately severe epigastric tenderness was confirmed with symptoms of fever, nausea, vomiting and moderate abdominal pain.

The amylase and the lipase declined to baseline in a week.

She was discharged from the hospital with a low-fat diet for 3 weeks.

She didn’t complain any special discomfort.

CT scan of abdomen showed a basically normal finding one month after discharge.

She felt pain at the transplant kidney area and the blood pressure dropped to 88/61 mmHg. Emergency ultrasound showed two huge hematoma around the graft.

Blood routine was remarkable for leukocytosis. Serum lipase was 156 U/L. Serum amylase was 424U/L. Amylase of drainage was 554 U/L.

Repeated CT showed acute pancreatitis (grade D on Balthazar score, no necrosis visible without contrast injection). Abdominal ultrasound showed no biliary duct dilatation.

A 48-year-old woman developed a donor-derived infection after kidney transplantation, resulting in a ruptured graft renal artery, followed by peritoneal drainage, blood and urine culture infections. She had not drink alcohol at all and she had no history of hyperlipemia.

A 48-years-old female worker received a DCD kidney transplant for the treatment of ESRD.

Emergency treatment including transplant exploration, renal hematoma removal, renal vascular reconstruction and ureteral reimplantation were performed.
